# Exploring the Influence of Personality Traits on Response to Esketamine nasal spray in Treatment-Resistant Depression: A Real-World Study

**DOI:** 10.1192/j.eurpsy.2025.1342

**Published:** 2025-08-26

**Authors:** M. Marino, E. Briasco, R. Guglielmo, E. Cavanna, F. Schiavon, G. Giacomini, D. Malagamba, G. Martinotti, M. Amore, G. Serafini

**Affiliations:** 1 Department of Neuroscience, Rehabilitation, Ophthalmology, Genetics, Maternal and Child Health, Section of Psychiatry, University of Genoa; 2 Istituto di Ricovero e Cura a Carattere Scientifico Ospedale Policlinico San Martino; 3 Department of Mental Health and Addiction, Azienda Sanitaria Locale 3 (ASL3) Genova, Genoa; 4 Department of Neurosciences, Imaging and Clinical Sciences, Università degli Studi G. D’Annunzio, Chieti, Italy; 5 Psychopharmacology, Drug Misuse and Novel Psychoactive Substances Research Unit, School of Life and Medical Sciences, University of Hertfordshire, Hatfield, United Kingdom

## Abstract

**Introduction:**

Treatment-resistant depression (TRD) is a severe condition with substantial economic and social impacts, defined by a lack of response to two or more adequate antidepressant treatments. Esketamine, an NMDA receptor antagonist, acts as an antidepressant by modulating glutamatergic transmission. Some studies suggest that dopaminergic system activation is crucial for the antidepressant effect of (S)-Ketamine (*Jelen LA et al. J Psychopharmacol. 2021 Feb;35(2):109-123*). Recently, nasal spray esketamine (ESK-NS) has been approved for TRD by the EMA and FDA, showing high response rates in some studies (*Martinotti ed al. 2022 J. Affect. Disord. 319, 646–654*). However, while the efficacy of ESK-NS varies among patients, studies on predictive factors of response, especially clinical ones, are limited, and no research has yet examined personality traits as predictors of ESK-NS response.

**Objectives:**

In this multicenter study we aim to investigate correlations between personality traits and response to ESK-NS in a real-world sample of subjects with TRD.

**Methods:**

Eighteen patients with TRD were enrolled in two different centers in Genoa. Sociodemographic and clinical data were collected through semi-structured interviews. The Temperament and Character Inventory (TCI) was administered before therapy (T0) to assess personality traits, while the Hamilton Depression Scale (HAM-D) was used at T0 and three months (T3) to evaluate depression severity and treatment response.

**Results:**

The mean age of patients was 56 years with SD 11,3 years. 66,6 % of patients (n=12) were female. The enrolled patients were divided into two groups (responders 55,6% and non-responders 44,4%) as determined by a reduction of at least 50% in the HAM-D score from T0 to T3. In a multivariate analysis with TCI as the dependent variable and HAM-D severity at T0 as a covariate, a statistically significant difference (p=0.018) was found between the responders and non-responders groups in the TCI Reward Dependence (RD) subscale with higher values in responders compared to non-responders.

**Conclusions:**

Previous studies have examined the link between personality traits and antidepressant response, but this correlation with ESK-NS hasn’t been previously investigated. This study, for the first time, demonstrates the correlation between Esketamine responders and RD traits, even when accounting for depressive severity (HAM-D). This may be because individuals with high RD are more sensitive to positive reinforcement. If ESK-NS improves mood and rewarding experiences, these patients might benefit more quickly. Additionally, RD is linked to dopamine, and since ESK-NS influences dopaminergic pathways, those with high RD may respond better to the treatment. This finding supports earlier research that anhedonic features predict better responses in TRD patients (*Pettorruso et al. Psychiatry Res. 2023 Sep;327:115378*).

**Disclosure of Interest:**

M. Marino: None Declared, E. Briasco: None Declared, R. Guglielmo: None Declared, E. Cavanna: None Declared, F. Schiavon: None Declared, G. Giacomini: None Declared, D. Malagamba: None Declared, G. Martinotti Grant / Research support from: Janssen-Cilag, Consultant of: Janssen-Cilag, Speakers bureau of: Janssen-Cilag, M. Amore: None Declared, G. Serafini: None Declared

